# Egg quality traits, blood biochemical parameters and performance of laying hens fed diet included processed oak fruit

**DOI:** 10.1002/vms3.381

**Published:** 2020-10-22

**Authors:** Fatemeh Bekri, Mehran Torki

**Affiliations:** ^1^ Animal Science Department College of Agriculture and Natural Resources Razi University Kermanshah Iran

**Keywords:** blood biochemicals, egg quality traits, laying hens, oak fruit, performance

## Abstract

To investigate the effects of replacing maize with various levels of raw and processed oak fruit in diet on productive performance of laying hens and egg quality traits, the total number of 168 Bovans white laying hens (30‐week age) were randomly distributed between 28 replicate cages and assigned to 7 experimental diets. Based on a 2 × 3 factorial arrangement of treatments including two dietary levels (10% and 20%) of raw, soaked or boiled oak fruit as well as a corn–soybean meal‐based diet, 7 experimental diets with 4 replicates and 6 chickens per replicate cages were evaluated during an 8‐week period. The data were analysed using GLM procedure of SAS. Significantly higher feed consumption was observed in 10% boiled oak compared with soaked oak (*p* < .05). Significantly improved feed conversion ratio was observed in 10% boiled oak compared with soaked oak (*p* < .05). Diet inclusion of 10% oak fruit caused significant increased shell weight. Significant interaction between oak level and processing method on the egg‐specific gravity was observed (*p* < .05), and higher egg‐specific gravity was detected in hens fed the diets included 20% boiled or 20% raw oak compared to 20% soaked oak. Significantly increased blood LDL level was observed in hens fed the diets that included boiled and soaked oak (*p* < .05). In conclusion, based on the results of the present study, 10% boiled oak can be substituted corn in diet of laying hens with no unfavourable effect on performance.

## Graphical abstract

Effects of dietary replacement of maize with various levels of raw and processed oak fruit on productive performance of laying hens and egg quality traits were investigated. Diet inclusion of 10% boiled oak as a substitute of corn could have a positive effect on performance of laying hens.

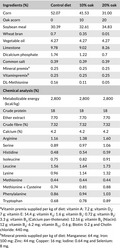

## 1 INTRODUCTION

Corn is the most important and traditional energy source in poultry diets; however, due to insufficient domestic production, a huge amount of corn is annually imported to Iran. Hence, finding new, local, unconventional feedstuffs are important. Oak acorn (*Quercus brantii* Lin.) is native tree in Iran (Zagross region) and annually large amounts of acorns (seed) are produced. This fruit contains high levels of starch (47%–60%) and 7%–14.4% lipids (Bouderoua et al., 2009), and due to its low price, it could be used as an alternative to corn in poultry diets (Bouderoua et al., 2009; Houshmand et al., 2015; Saeidi et al., 2017). Lipids of oak acorns contain linoleic acid and ω‐3 polyunsaturated fatty acid (Petrovic et al., 2004). Oak acorns (OA) contain bioactive anti‐nutritive factors, including tannins (7%), which possess antioxidant activity (Rakić et al., 2007). Tannins are water‐soluble polyphenolic compounds, which reduce diet palatability and feed intake (Ghaedi et al., 2018), pancreatic hypertrophy, protein and starch digestibility (Mahmood et al., 2007, 2008). High level of OA in diet may be toxic and reduce product and egg hatchability in laying hens (De Boer & Bickel, 1988). Various strategies, such as chemical treatment (Medugu et al., 2012), water or alkali, acetic acid, de‐hulled and sodium hydrogen carbonate distilled water (Mahmood et al., 2008), have been used to alleviate the anti‐nutritive consequences of tannins. Few studies have been conducted to investigate the influence of diet inclusion of OA on productive performance of laying hens and egg quality traits. The purpose of this trial was to study the effects of diet inclusion of raw and processed OA on productive performance of laying hens, some blood biochemical parameters and egg quality traits.

## 2 MATERIALS AND METHODS

### 2.1 Animals, management and sample collection

The total number of 168 Bovans white laying hens (30‐week age) were randomly distributed between 28 replicate cages and assigned to 7 experimental diets. Based on a 2 × 3 factorial arrangement of treatment including two diet levels (10 and 20 %) of raw, soaked or boiled oak fruit as well as a corn–soybean meal‐based diet as control, 7 iso‐caloric and iso‐nitrogenous diets (Table 1) with 4 replicates and 6 chickens per replicate cages were formulated and assigned to feed laying hens during an 8‐week experimental period.

**TABLE 1:** Composition and chemical analyses of experimental diets

Ingredients (%)	Control diet	10% oak	20% oak
Corn	52.07	41.53	31.00
Oak acorn	0	10	20
Soybean meal	30.39	32.61	34.83
Wheat bran	0.7	0.35	0.01
Vegetable oil	4.27	4.27	4.27
Limestone	9.78	9.02	8.26
Dicalcium phosphate	1.74	1.22	0.7
Common salt	0.39	0.39	0.39
Mineral premix^a^	0.25	0.25	0.25
Vitaminpremix^b^	0.25	0.25	0.25
DL‐Methionine	0.16	0.11	0.05

Chemical analysis (%)			
Metabolizable energy (kcal/kg)	2,800	2,800	2,800
Crude protein	18	18	18
Ether extract	7.70	7.70	7.70
Crude fibre (%)	7.32	7.32	7.32
Calcium (%)	4.2	4.2	4.2
Arginine	1.16	1.38	1.60
Serine	0.89	0.97	1.06
Histidine	0.48	0.54	0.59
Isoleucine	0.75	0.82	0.91
Leucine	1.56	1.64	1.73
Lysine	0.96	1.14	1.32
Methionine	0.44	0.44	0.44
Methionine + Cysteine	0.74	0.81	0.88
Phenylalanine	0.86	0.94	1.03
Tryptophan	0.68	0.78	0.89

^a^ Vitamin premix supplied per kg of diet: vitamin A: 7.2 g; vitamin D_3_: 7 g; vitamin E: 14.4 g; vitamin K_3_: 1.6 g; vitamin B_1_: 0.72 g; vitamin B_2_: 3.3 g; vitamin B_3_ (Calcium pan‐thotenate): 12.16 g; vitamin B_5_ (Niacin): 12 g; vitamin B_6_: 6.2 mg; vitamin B_12_: 0.6 g; Biotin: 0.2 g and Cholin chloride: 440 mg.

^b^ Mineral premix supplied per kg of diet: Manganese: 64 mg; Iron: 100 mg; Zinc: 44 mg; Copper: 16 mg; Iodine: 0.64 mg and Selenium: 8 mg.

### 2.2 Oak fruit preparation and processing

The adequate amount of acorn as whole fruits was collected from the forests of Ilum, the west part of Iran. The seed was dried in shade after removing seed coat. In the soaking processing method, acorns were weighted, and based on the previous report (Ghaderi‐Ghahfarrokhi et al., 2017), soaked in distilled water for 10 hr and then dried for 72 hr under the sun. In the boiling method, acorns were weighted, boiled within distilled water for 1.5 hr and then put under the sun for 72 hr (Vijayakumari et al., 1998). Approximate analysis of OA was done and DM, EE, CF, CP, Ash, tannins and NFE were analysed according to the AOAC (1995) procedure (Table 2). Feed intake (FI) was recorded weekly, whereas egg production (EP) and egg weight (EW) were recorded daily. Average daily feed intake (ADFI), egg mass (EM), hen‐day egg production (HDP) and feed conversion ratio (FCR) were calculated. At 38 weeks of age, blood samples (2.5 ml) were collected, after an overnight feed deprivation, via the brachial vein of one birds per each replicate cages, and centrifuged for 15 min at 1008 *g* to obtain serum samples. To determine the blood levels of total protein, cholesterol, triglyceride, high density lipoprotein (HDL) and glucose contents, sera were analysed via spectrophotometer (Abbott alcyon 300, USA) by using Pars‐Azmon kits (Tehran, Iran).

**TABLE 2:** Proximate analysis of oak fruit (% DM)

Component	DM^a^	Ash	CP^b^	EE^c^	CF^d^	Tannin	Phenolic compounds
Raw oak	88.74	1.4	4.19	8.36	2.34	5.60	6.24
Soaked oak	90.21	1.9	6.95	9.12	2.19	4.14	4.69
Boiled oak	93.61	1.11	7.15	9.98	2.10	3.88	4.53

^a^ Dry matter.

^b^ Crude protein.

^c^ Ether extract.

^d^ Crude fibre.

To measure egg quality characteristics including albumen and yolk indexes, Haugh unit (HU) and shell weight, four eggs per each replicate (16 eggs/treatment) were randomly selected. The collected eggs were broken after individually weighting and then the egg shells were washed and dried in an oven to determine shell weight. Albumen and yolk heights and widths were measured for HU and yolk indexes. Then, HU was calculated using the following formula: HU = 100 log (AH + 7.57 − 1.7 EW^0.37^), where: AH: albumen height (mm), EW: egg weight (g).

### 2.3 Chemical analysis of feed

Formulated and prepared diets were initially analysed to determine dry matter (DM), crude protein (CP), crude fibre (CF), ether extract (EE) and ash using AOAC (Table 2).

### 2.4 Statistical analyses

Data were analysed using SAS 9.1. The statistical model for performance data was as follows: *Y_ijk_* = *µ* + *A_i_* + *B_j_* + *AB_ij_* + e*_ijk_*_,_ where: *Y_ij_* is observations, *μ* is overall mean, *A_i_* is effect of treatments or oak level, *B_j_* is effect of processing method, *AB_ij_* is the interaction between *A_i_* and *B_j_* (*A_i_* × B_j)_ and e_ijk_ is residual error. Means were compared by Duncan’s multiple range tests. Treatments differences were considered significant at *p* < .05. Significantly different means were separated by orthogonal contrast tests.

## 3 RESULTS

### 3.1 Performance of laying hens

Performance of laying hens (30–38 weeks of age) is presented in Table 3. The highest egg mass (EM), egg production (EP) and egg weight (EW) were observed in hens fed 10% oak‐included diet (*p* < .05). Level of oak in diet and processing method had no significant effect on FI during 30–38 weeks of age. Processing method and dietary level of oak had significant effects on FCR during various experimental phases (30–34, 34–38 and 30–38 weeks of age). During 30–34 weeks of age, the best and the worst FCR were observed in 20% and 10% oak groups, respectively. Decreased FCR were observed in 10% oak groups during 34–38 and 30–38 weeks of age (*p* < .05). Improved FCR were observed in birds fed the boiled and raw oak included diet during 34–38 and 30–38 weeks of age, (*p* < .05). The higher EM, EP and EW were seen in the birds fed processed oak‐included diet compared with those fed raw oak‐included diet (*p* < .05); however, the same trend was not detected during 30–34 weeks of age. Increased FI and decreased FCR were observed in hens fed 10% to 20% raw oak‐included diets during 34–38 weeks of age (Table 3). The results of running the orthogonal comparison for performance traits (EP, EM, FI and FCR) and egg shell weight were shown in Tables 4 and 5. Feeding 10% raw oak caused higher EM and EP compared to 20 raw oak (*p* < .05).

**TABLE 3:** Effect of different levels of raw and processed oak fruit on performance of laying hens

Treatments	Feed intake (g f/hen/day)				FCR		Egg mass (g egg/h/d)			Egg production (%)			Egg weight (g)		
Weeks	30–34	34–38	30–38	30–34	34–38	30–38	30–34	34–38	30–38	30–34	34–38	30–38	30–34	34–38	30–38
Oak (%)															
10	109.5	109.8^a^	109.6	2.5^a^	2.0^b^	2.1^b^	51.2^a^	53.0^a^	52.1^a^	89.6^a^	89.7^a^	89.7^a^	51.2^a^	53.0^a^	52.1^a^
20	109.0	1.9.2^b^	109.1	2.1^b^	2.4^a^	2.4^a^	44.9^b^	44.1^b^	44.9^b^	81.0^b^	78.5^b^	79.8^b^	44.9^b^	44.1^b^	44.9^b^
*SEM* *	0.26	0.11	0.17	0.07	0.04	0.05	1.34	1.06	1.14	2.37	2.00	2.10	1.34	1.06	1.14
Processing															
Raw	109.5	109.2^b^	109.3	2.2^b^	2.3^ab^	2.2^b^	49.7^a^	48.3^b^	49.0^ab^	87.9^a^	82.1^a^	85.4^ab^	49.7^a^	48.3^b^	49.0^ab^
Soaking	109.0	109.5^a^	109.2	2.6^a^	2.3^a^	2.4^a^	43.6^b^	47.2^b^	45.4^b^	76.9^b^	80.8^b^	78.9^b^	43.6^b^	47.2^b^	45.4^b^
Boiling	109.3	109.8^a^	109.5	2.1^b^	2.1^b^	2.1^b^	50.8^a^	51.5^a^	51.2^a^	91.1^a^	88.6^a^	89.9^a^	50.8^a^	51.5^a^	51.2^a^
*SEM*	0.32	0.14	0.21	0.09	0.06	0.07	1.64	1.30	1.39	2.91	2.44	2.58	1.65	1.29	1.39
*p*‐Value															
O	0.26	0.006	0.07	0.009	0.0001	0.0009	0.003	0.0001	0.0003	0.02	0.0009	0.003	0.003	0.0001	0.0003
P	0.57	0.03	0.65	0.009	0.04	0.02	0.01	0.04	0.02	0.007	0.009	0.02	0.01	0.04	0.02
O × P	0.86	0.12	0.93	0.32	0.45	0.49	0.51	0.42	0.70	0.56	0.75	0.88	0.51	0.42	0.70

The existence of similar letters indicates that there is no significant difference.

FCR, feed conversion ratio; O, oak; P, processing.

* Pooled standard error of the mean.

**TABLE 4:** Effect of different levels of raw and processed Oak fruit on egg quality traits of laying hens

Treatments	Shell weight (g)	Yolk weight (g)	White weight (g)	Shell thickness (mm)	Shape index (%)	Yolk index (per cent)	Yolk colour (Roch)	Haugh unit	Special Weight
Oak (%)									
10	6.0^a^	16.5	34.4	39.4	74.9	42.0	4.3	95.7	1.0
20	5.6^b^	16.6	33.4	38.4	75.6	43.7	4.3	94.8	1.0
*SEM* *	0.15	0.3	0.4	0.63	0.50	0.88	0.14	1.14	1.28
Processing									
Raw	5.9	33.9	34.0	39.2	74.4	43.0	4.5	95.6	1.09
Soaking	5.9	34.3	33.9	38.6	76.5	41.1	4.4	94.1	1.08
Boiling	5.8	34.0	34.3	38.8	74.9	43.6	4.0	96.0	1.09
*SEM*	0.18	0.4	0.5	0.78	0.60	1.08	0.18	1.40	0.001
CV	9.06	6.91	4.22	5.68	2.30	7.16	12.14	4.18	0.48
*p*‐Value									
O	0.04	0.93	0.33	0.28	0.34	0.20	0.84	0.60	0.44
P	0.86	0.43	0.80	0.86	0.06	0.57	0.14	0.60	0.37
O × P	0.09	0.43	0.58	0.16	0.64	0.15	0.27	0.03	0.004

The existence of similar letters indicates that there is no significant difference.

O, oak; P, processing.

* Pooled standard error of the mean.

**TABLE 5:** Interaction of treatments on specific gravity and Haugh unit

Treatments	Special weight	Haugh unit
Control	1.09^abc^	93.9^ab^
10% raw oak	1.08^bc^	95.7^ab^
10% soaked oak	1.09^abc^	97.5^a^
10% boiled oak	1.09^ab^	93.8^ab^
20% raw oak	1.10^a^	95.4^ab^
20% soaked oak	1.08^c^	90.7^b^
20% boiled oak	1.08^bc^	98.3^a^
*SEM* *	0.002	1.91

The existence of similar letters indicates that there is no significant difference.

* Pooled standard error of the mean.

### 3.2 Egg quality

Effects of diet inclusion of raw and processed oak on egg quality characteristics were presented in Table 6. Increased shell weight was detected in 10% oak group (*p* < .05). No significant difference in egg specific gravity was observed among treatments. The interaction between diet inclusion of oak and processing on egg‐specific gravity was significant (*p* < .05); the higher level was seen in hens fed raw oak‐included diet. As it is shown in Table 7, increased HU was detected in laying hens fed 20% boiled oak and 10% soaked oak. The higher egg special weight was observed in 20% raw oak (*p* < .05).

**TABLE 6:** Orthogonal comparisons of treatments for egg mass and egg production

Treatments	Egg mass				Egg production			
Week	34–38		30–38		34–38		30–38	
Orthogonal contrasts	$\bar{x}$	*p*‐Value	$\bar{x}$	*p*‐Value	$\bar{x}$	*p*‐Value	$\bar{x}$	*p*‐Value
Control vs. other treatments	96.3 vs. 80.9	0.002	95.5 vs. 84.7	0.007	57.8 vs. 49.0	0.0001	56.5 vs. 48.5	0.0006
10% R vs. 20% R	90.0 vs. 75.9	0.005	90.1 vs. 80.8	0.06	53.6 vs. 43.0	0.0003	52.8 vs. 45.3	0.009
10% P vs. 20% P	89.8 vs. 75.8	0.006	89.5 vs. 79.3	0.006	52.8 vs. 45.9	0.0008	51.8 vs. 44.8	0.001
10,20% R vs. 10,20% P	82.1 vs. 84.7	0.53	85.4 vs. 84.4	0.71	48.3 vs. 49.4	0.49	49.0 vs. 48.3	0.65

O, oak; P, processing.

**TABLE 7:** Effect of different levels of raw and processed oak fruit on blood biochemicals of laying hens

Treatments	Glucose (mg/dl)	Triglyceride (mg/dl)	Total protein (g/dl)	Cholesterol (mg/dl)	HDL (mg/dl)	LDL (mg/dl)
Oak (%)						
10	133.9	173.1	4.6	147.2	41.2	67.1
20	144.2	168.1	4.0	140.5	46.1	54.8
*SEM* *	14.62	10.86	0.25	11.78	1.74	8.9
Processing						
Raw	154.8	183.1	4.7	124.3	44.2	41.0^b^
Soaking	125.3	172.9	4.4	164.9	43.3	81.4^a^
Boiling	136.9	155.9	3.7	142.3	43.5	60.4^ab^
*SEM*	17.91	13.30	0.30	14.43	2.14	10.97
CV	36.43	22.06	20.30	28.39	13.89	50.92
*p*‐Value						
O	0.6	0.7	0.11	0.6	0.06	0.34
*p*	0.5	0.3	0.08	0.1	0.94	0.05
O × P	0.8	0.8	0.18	0.6	0.18	0.18

The existence of similar letters indicates that there is no significant difference.

HDL, high‐density lipoprotein; LDL, low‐density lipoprotein; O, oak; P, processing.

* Pooled standard error of the mean.

### 3.3 Blood parameters

Effects of diet inclusion of raw and processed oak on blood biochemical parameters including glucose, triglyceride, total protein, cholesterol, HDL and LDL in laying hens were presented in Table 8. Significantly decreased level of blood LDL was detected in birds fed raw oak‐included diet compared with the other groups (*p* < .05). The results of orthogonal comparisons of dietary treatments in terms of feed intake, feed conversion ratio and shell weight were shown in Table 8. Improved FCR and shell weight were observed in hens fed 10% processed oak‐included diet compared with those fed raw oak‐included diet during 34–38 and 30–38 weeks of age.

**TABLE 8:** Orthogonal comparisons of treatments for feed intake, feed conversion ratio and shell weight

Treatments	Feed intake				FCR				Shell weight			
Week	34–38		30–38		34–38		30–38		34–38		30–38	
Orthogonal contrasts	$\bar{x}$	*p*‐Value	$\bar{x}$	*p*‐Value	$\bar{x}$	*p*‐Value	$\bar{x}$	*p*‐Value	$\bar{x}$	*p*‐Value	$\bar{x}$	*p*‐Value
Control vs. other treatments	109.6 vs. 109.5	0.72	109.4 vs. 109.4	0.96	1.1 vs. 2.3	0.02	1.9 vs. 2.2	0.0007	1.9 vs. 2.3	0.003	6.0 vs. 5.8	0.46
10% R vs. 20% R	109.7 vs. 108.7	0.001	109.6 vs. 109.0	0.21	2.1 vs. 2.2	0.33	2.0 vs. 2.5	0.001	2.1 vs. 2.4	0.03	5.9 vs. 5.8	0.89
10% P vs. 20% P	109.8 vs. 109.5	0.20	109.6 vs. 109.2	0.20	2.1 vs. 2.5	0.005	2.1 vs. 2.4	0.001	2.1 vs. 2.5	0.001	6.2 vs. 5.5	0.01
10,20% R vs. 10,20% P	109.1 vs. 109.6	0.02	109.3 vs. 109.4	0.85	2.2 vs. 2.4	0.14	2.3 vs. 2.2	0.67	2.2 vs. 2.3	0.45	5.9 vs. 5.8	0.85

Abbreviations: FCR, feed conversion ratio; FI, feed intake; O, oak; P, processing.

## 4 DISCUSSION

Very limited publications regarding the effects of diet inclusion of oak on performance of laying hens can be found in the literature. Increased feed intake of hens fed 10% boiled oak in the current investigation is in agreement with the findings of Ghaderi‐Ghahfarrokhi et al., (2017), who reported that boiling oak for 60 min or autoclaving for 30 min reduce polyphenol content of oak. In addition, increased feed intake due to decreasing polyphenol content of oak was observed in soaking oak compared with raw oak (Deshpande et al., 1982). No significant effect of dietary oak inclusion on feed intake of laying hens during 30 to 38 weeks of age is in agreement with the finding of Saffarzadeh et al. (1999), who reported no significant effect of diet inclusion of acorn seed (0, 10, 20 and 30%) on feed intake, mortality rate and body weight. They also observed improved egg production rate, egg mass and feed efficiency. Houshmand et al. (2015) reported increased feed intake of broilers fed oak‐included diet. Improved FCR in the hens fed 10% oak‐included diet is in agreement with the observations by Saeidi et al. (2017), who fed broiler oak‐included diet with or without polyethylene glycol. In a study conducted by Sharif et al. (2012), water treatment reduced the tannin content of sorghum and improved feed efficiency of broilers.

In the current study, increased EM, EP and EW were observed in laying hens fed 10% boiled oak‐included diet, which are in agreement with Saffarzadeh et al., (2000) who showed improved egg weight, egg production rate and egg mass of laying hens fed oak‐included diet during the first production phase. Kargar et al. (2016) reported that using various levels of raw and processed oak had no significant effect on egg mass in laying hens fed diet included raw and processed oak. Ghaedi et al. (2018) reported that feeding oak‐included diet significantly increased egg shell in laying hens. The negative effects of tannins in raw oak acorn on minerals (Ca, P, Mg, Na, K, Fe and Co) availability and tibia bone have been reported by Hassan et al. (2003). Using processed oak acorn in broiler diet had beneficial effects on performance (Bouderoua et al., 2009). In the current investigation, egg quality traits such as yolk weight, shell thickness, shape index, yolk index and yolk colour were not negatively affected by including diet with raw and processed (boiled or water‐soaked) oak seed. Decreased egg shell weight was observed in the hens fed 20% oak‐included diet compared to 10%, which may be partly due to low tannins at 10% oak level that in turn increased feed intake and nutrients availability (Laudadio & Tufarelli, 2010). In the current study, egg special weight and Haugh unit were significantly increased by diet inclusion of oak compared with control. Chen et al., (2018) reported the increased Haugh unit in laying hens fed sorghum‐included diet compared with the control, which may be partly due to increased nutrient digestion (Ebadi et al., 2005).

Based on the results of current study, no significant effect of treatment was observed on the blood levels of glucose, triglyceride, total protein, cholesterol, HDL and LDL, which is in consistent with Rezaei and Semnaninejad (2016), who reported no significant effect of dietary raw and processed oak acorn on serum glucose, haemoglobin, albumin and total protein concentrations. However, these findings are in contrast with the data reported by Bouderoua and Selselet‐Attou (2003) that may be due in part to the oak species and the lower diet inclusion level of oak acorn in the present trial (10% and 20%) compared with the level they used (60%). In the current study, decreased blood level of LDL was detected in hens fed water‐soaked oak‐included diet.

## 5 CONCLUSION

Overall, based on the results of the current study, diet inclusion of 10% boiled oak as a substitute of corn could have a positive effect on performance of laying hens.

## CONFLICT OF INTEREST

The authors have no conflict of interest to declare.

## ANIMAL WELFARE STATEMENT

The authors confirm that the ethical policies of the journal, as noted on the journal’s author guidelines page, have been adhered to and the appropriate ethical review committee approval has been received. The authors confirm that they have followed EU standards for the protection of animals used for scientific purposes.

### PEER REVIEW

The peer review history for this article is available at https://publons.com/publon/10.1002/vms3.381.
